# Plant-Based Burgers Made with Green Banana Biomass (GBB) Associated with Teff and Chickpea Derivatives

**DOI:** 10.3390/foods14101782

**Published:** 2025-05-17

**Authors:** Ziane da Conceição das Mercês, Natalia Maldaner Salvadori, Sabrina Melo Evangelista, Tatiana Barbieri Cochlar, Cristine da Silva Medeiros, Rafaela Giuliana Hermelino Lima, Amanda Soares Bandeira, Ana Karolina Fortunato de Souza, Alessandro de Oliveira Rios, Viviani Ruffo de Oliveira

**Affiliations:** 1 Posgraduate Program in Food, Nutrition and Health, Federal University of Rio Grande do Sul (UFRGS), Porto Alegre 90035-003, RS, Brazil; zianemerces@gmail.com (Z.d.C.d.M.); natisalvadori18@gmail.com (N.M.S.); sabrina.evangelista@ufrgs.br (S.M.E.); tatianabarbieri2010@hotmail.com (T.B.C.); crikamedeiros@gmail.com (C.d.S.M.); 2 Nutrition Course, Federal University of Rio Grande do Sul (UFRGS), Porto Alegre 90035-003, RS, Brazil; rafaghl@hotmail.com (R.G.H.L.); amanda.soares.bandeira@gmail.com (A.S.B.); anakarolinatcc@gmail.com (A.K.F.d.S.); 3 Institute of Food Science and Technology, Federal University of Rio Grande do Sul (UFRGS), Porto Alegre 91501-970, RS, Brazil; alessandro.rios@ufrgs.br; 4 Department of Nutrition, Federal University of Rio Grande do Sul (UFRGS), Porto Alegre 90035-003, RS, Brazill

**Keywords:** meat substitutes, *Eragrostis tef*, meat analogs, *Musa* spp., burger analogs

## Abstract

The objective of this study was to develop meat analog burgers using green banana biomass (GBB) combined with teff and chickpea derivatives, as well as to evaluate their chemical and technological quality, in addition to comparing them with industrialized meat and plant-based burgers. Four formulations (F1: 100% GBB; F2: 75% GBB; F3: 60% GBB; and F4: 50% GBB, with 25% teff and 25% chickpeas) were developed and compared to the following industrialized burgers: F5 (meat-based) and F6 (plant-based). All the samples were subjected to physical (initial/final weight, diameter, height, color, and texture) and chemical (pH, proximate composition, fiber, and caloric value) analyses. Among the treatments, F4 showed the highest initial weight (223.00 g) and final weight (201.66 g), initial diameter (12.33 cm) and final diameter (11.96 cm), and a reduction in height from 2.04 cm to 1.57 cm. In terms of firmness, F4 was significantly higher than that of the other treatments (*p* ≤ 0.05). Regarding its chemical composition, F4 presented a high protein content (10.25%) and energy value (285.30 kcal). The total fiber content for F1 to F4 was 3.62%, 3.74%, 3.97%, and 4.15%, respectively, while F6 (plant-based) reached 5.69%. These findings indicate that the combination of GBB with teff and chickpeas, especially in F4, was favorable for producing meat analog burgers with promising technological and nutritional properties.

## 1. Introduction

The creation of new food products has increasingly turned to sustainable approaches, as in the case of plant-based burgers or meat analogs. These products not only contribute to human nutrition, offering chemical and technological quality, but also have easy reproducibility [1]. These plant-based alternatives represent an innovation in the contemporary food and culinary industry [2,3,4]. Being designed to resemble meat in their appearance, flavor, and texture, they can be completely free of meat or contain small amounts of this ingredient [5].

Among meat products, beef burgers stand out as one of the most commonly consumed globally [6]. Against this background, plant-based burgers are emerging as a promising alternative, simulating the flavor and physico-chemical properties of a traditional hamburger. Their formulation usually includes textured soy as a substitute for animal protein, as well as whole grains and plant oils as a source of lipids, providing characteristics like those of a conventional beef burger [7].

Banana (*Musa* spp.) is a highly nutritious, low-cost food that is easy to find and can be consumed in its entirety [8]. This green fruit has attracted the attention of the consumer market due to its nutritional value. Its chemical composition also includes phytosterols, phenolic compounds, and a strong antioxidant capacity [9,10]. Green banana biomass (GBB) is a product from the processing of bananas, obtained by cooking green bananas. The product has also been described as a dough, with a high content of resistant starch and a low content of sugars and aromatic compounds [11]. Because of its tasteless and odorless characteristics, it can be used in food production as a thickener and emulsifier [9]. Some important nutrients are also present in relevant concentrations, such as phenolic compounds, fiber, mineral salts, and B vitamins (B1 and B6) [9,10,12].

Teff (*Eragrostis tef*) is an important annual food crop native to Ethiopia and Eritrea, serving as a staple for producing a variety of traditional foods and beverages [13]. Teff is considered a promising source of gluten-free flour. The products of its starch hydrolysis (simple sugars) and protein hydrolysis (amino acids) are essential for producing fermented beverages and baked goods [14,15,16]. The regular intake of teff products helps minimize the risk of anemia because teff is a good source of iron and calcium [14,17,18].

Chickpeas (*Cicer arietinum* L.), in addition to being an important source of fiber, carbohydrates, vitamins, and minerals, also consist of phytochemical compounds, such as carotenoids, phenolics, and isoflavones [19]. It is also considered an important legume due to its good components. Chickpea flour has been used in different meat products and as an analog to meat to improve its nutritional values [20].

Although green banana biomass (GBB), teff, and chickpeas have already been added to some food products and have shown functional and technological potential, no research has evaluated these ingredients in combination in formulations of meat analog burgers. Thus, there is still a gap in the physico-chemical and technological evaluation of these alternative formulations, especially for varying proportions of GBB.

The objective of this paper was to develop meat analog burgers using green banana biomass (GBB) combined with teff and chickpea derivatives, as well as to evaluate their chemical and technological quality, in addition to comparing them with industrialized meat and plant-based burgers.

## 2. Materials and Methods

### 2.1. Acquisition of Ingredients and Preparation of Meat Analog Burgers

All ingredients used in the burgers were purchased from grocery stores in the city of Porto Alegre, RS, Brazil. The meat analog burgers were prepared in the Dietetics Laboratory of the Medicine Faculty at the University of Rio Grande do Sul-UFRGS.

Table 1 presents all the ingredients used for the formulation of the burgers, which included the following: green banana biomass (GBB), teff and chickpeas, textured soy protein, chickpea flour, aquafaba, salt, ground black pepper, garlic, onion, fresh green seasoning (chives + parsley), dehydrated complete seasoning, and crushed ice. The percentages of green banana biomass (GBB) were different and were gradually reduced to integrate the ingredients. Aquafaba, soy protein, cooked chickpeas, spices, and seasonings were kept constant in all formulations.

Different formulations were thoroughly tested previously, including the treatment containing 25% biomass and 75% teff and chickpeas, but treatments that were not considered promising or optimal were not included in the final analyses. For example, those with an excessively firm texture and a tendency to break would have made their technological evaluation unfeasible.

The formulations were adapted from the study by De Oliveira Rosa and Lobato [21]. The ingredients were weighed using a 0.01 g digital analytical scale (model UX-6200H, Unibloc, Shimadzu^®^, Kyoto, Japan) duly calibrated and certified by the standards established by Inmetro-National Institute of Metrology, Quality, and Technology [22].

### 2.2. GBB Making

GBB was prepared according to a methodology adapted from Lovis et al. [23] and Martins et al. [24]. Bananas (*Musa sapientum*) cv. “Prata” were completely green according to the ripening scale, as shown in Von Loesecke [25].

The elaboration of the GBB began by sanitizing the bananas in a 200 ppm chlorine solution. Then, the bananas were rinsed under running water and subjected to a pressure-cooking process for 8 min, counted from the beginning of pressure, using a domestic pressure cooker at a temperature of approximately 200 °C in a conventional Brastemp^®^ stove 4-burner model, Joinville, Brazil. After cooking, manual peeling was performed, followed by the processing of the cooked banana using a 3-in-1 mixer, Turbo Processor Philco^®^, Curitiba, Brazil.

### 2.3. Making and Molding of the Plant-Based Burgers

**Figure 1:**
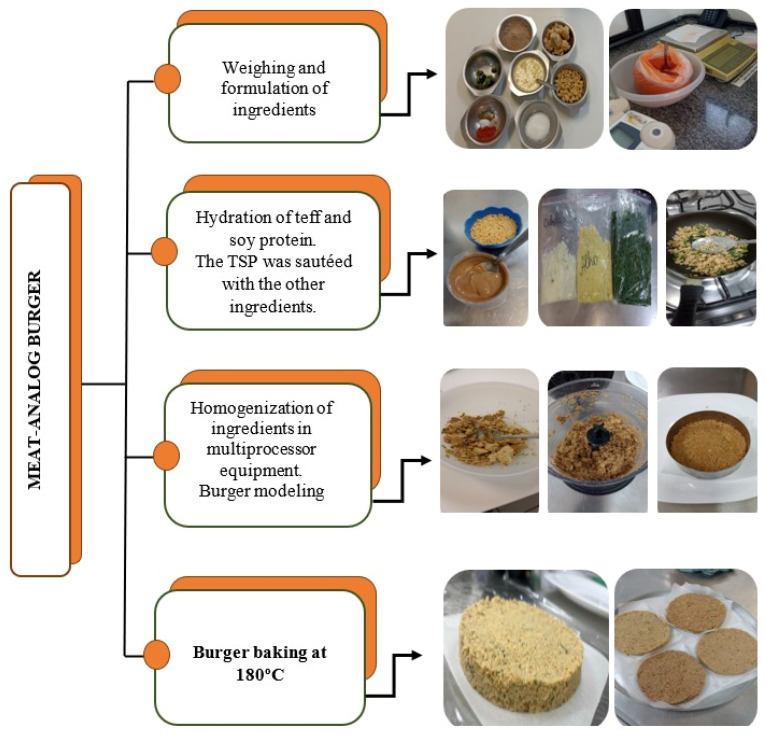
Flowchart for the preparation of green banana analog burgers with biomass, meat, plant-based flours, and chickpeas. Source: study data.

Burger-making began by weighing the ingredients on a 0.01 g analytical scale (Unibloc, Shimadzu^®^, model UX-6200H, Kyoto, Japan). After homogenizing the ingredients in the Philco 3 In 1 Turbo Processor Mixer, they were manually molded with a stainless-steel rim to create a 12 × 3 burger and baked in a conventional oven preheated at 180 °C with Brastemp^®^ model with 4 burners for 10 min. The step-by-step elaboration of the hamburgers is shown in Figure 1. In addition to the production of the burgers, two industrialized burgers were also evaluated for comparison in physical and chemical analyses: F5—industrialized/meat burger; F6—industrialized/plant-based burger.

### 2.4. Physical Analyses

#### 2.4.1. Weight

All samples had their initial and final weight measured in triplicate using a 0.01 g analytical scale (Unibloc, Shimadzu^®^, model UX-6200H, Kyoto, Japan).

#### 2.4.2. Diameter

All samples had their mean diameters determined by measuring the cross-section at three points of the samples and in triplicate using a universal metallic caliper (150 M, 0.02 mm pitch, Mtx^®^). After the baking process, the diameters were also determined. 

#### 2.4.3. Height

The height of the samples was determined in triplicate measurements at three different points using a Mtx^®^ universal metal caliper (150 mm, 0.02 mm pitch, China). After the baking process, the height was determined likewise.

#### 2.4.4. Water Retention Capacity–WRC

WRC was calculated as described in the studies by Roque-Specht et al. [26], where it is calculated based on the difference between the water content of the raw sample and the water content retained after baking, as shown in the following formula:

WRC (%) = (Moisture retained after baking) ×100

Moisture begins.

#### 2.4.5. Color

The color of the burgers was assessed after baking, in triplicate, using a Konica Minolta^®^ colorimeter, model CR-400, Kyoto, Japan, following the CIELAB methodology. The parameters of luminosity (L*) and chromaticity (a* and b*) were determined. L* ranges from 0 to 100 and indicates the brightness of the sample—the higher the value, the lighter the color. The a* value expresses the hue between green (negative values) and red (positive values), while b* represents the variation between blue (negative values) and yellow (positive values).

All color measurements were taken after baking. In the ΔE equation, L0*, a0*, and b0* correspond to the color parameters of the reference formulation (F1), while L*, a*, and b* refer to the other samples compared. The total color difference (ΔE) was calculated according to Kim et al. [27] using the following equation:

ΔE = [(L0* − L*)2 + (a0*–a*)2 + (b0* − b*)2]1/2

where the following apply:

L0* and L* = represent the luminosity (L*) values of the treatments;

a0* and a* = are the values of the green-red coordinate (a*);

b0* and b* = are the values of the blue-yellow coordinate (b*).

#### 2.4.6. Texture

The texture parameters of treatments F1—100%, F2—75%, F3—60%, F4—50%, F5—industrialized/meat, and F6—industrialized/plant-based were measured in quintuplicate using a Stable Micro Systems^®^ texturometer (model TA. XT plus, Surrey, United Kingdom) and 36 mm cylindrical probe (code P/36R).

The analyses were performed via penetration, determining the maximum force in the first compression cycle under the following conditions: maximum speed of 4.0 mm/s, minimum speed of 0.01 mm/s, and rupture distance of 0.001 mm with three repetitions. Based on the results from the texturometer, the rheological parameters, such as firmness, resilience, elasticity (ratio between two deformations), cohesiveness, chewiness, and gumminess, were calculated [28].

### 2.5. Chemical Analysis

#### 2.5.1. Sample Preparation

After the burgers were made, they were baked in an oven and then chemically analyzed. Due to the consistency of the products, it was necessary to store the samples in sealed plastic packaging and freeze them in an Electrolux^®^ Freezer (Curitiba, Brazil), model FE22 (173L), at a temperature of −18 °C.

#### 2.5.2. Proximate Composition and Caloric Values

The samples were analyzed to determine moisture, ash, protein, lipid, and carbohydrate content according to the procedure described by the Association of Official Analytical Chemists [29]. All analyses were performed in triplicate, and the values were expressed in g/g of wet matter.

The caloric values of the samples were calculated based on the results obtained for macronutrients (proteins, carbohydrates, and lipids) and expressed in kilocalories (kcal). The calculated values were based on Atwater system using the following formula:

Energy (kcal) = [Protein (g) × 4] + [Carbohydrate (g) × 4] + [Lipid (g) × 9]

where coefficients 4, 4, and 9 correspond to the average energy value (in kcal) provided per gram of protein, carbohydrate, and lipid, respectively.

#### 2.5.3. Soluble, Insoluble, and Total Fibers

The amount of fiber was determined in duplicate using the enzymatic gravimetric method described by the Association of Official Analytical Chemists (AOAC) [29]. The samples were digested with heat-resistant α-amylase, protease, and amyloglucosidase using the Sigma-Aldrich Kit (TDF100A-1KT), Darmstadt, Germany.

#### 2.5.4. Hydrogen Potential-pH

pH was determined using a potentiometric electrochemical method using a pH meter (Quimis^®^, model Q400AS, São Paulo, Brazil). The analyses were performed in triplicate on baked burgers [30].

### 2.6. Statistical Analysis

All the analyses were performed on all the treatments (F1 to F6). Physical and chemical analyses were carried out in triplicate, except for fiber analyses, which were performed in duplicate, and texture analyses, which were performed in quintuplicate. The data were subjected to one-way ANOVA followed by Tukey’s test at a significance level of 5%. SPSS software, version 29.0 [31], was used to analyze the data.

## 3. Results and Discussion

Table 2 shows the means and standard deviations of the physical analyses of all the treatments (F1, F2, F3, F4, F5, and F6), presenting detailed characteristics of the burgers through the physical results. The determination of initial and final weight (g), diameter (cm), and height (cm) aimed to evaluate the physical behavior of the burgers after baking, especially in relation to cooking loss (%) and diameter reduction (%). These parameters are important indicators of product quality and technological performance, especially in plant-based formulations that differ from meat in texture and water and oil retention.

The physical parameters of the burgers showed clear trends in response to the progressive reduction in green banana biomass (GBB) and the incorporation of teff and chickpeas. F4 (50% GBB) consistently showed the highest initial and final weight (223.00 ± 11.35 g and 201.66 ± 11.01 g, respectively; *p* ≤ 0.05), as well as the highest initial and final diameter (12.33 ± 0.56 cm and 11.96 ± 0.58 cm). These results suggested that the combination of GBB with teff and chickpeas contributed to better water-holding capacity and structural integrity during baking, minimizing weight loss and shrinkage.

The results for the initial and final height of the different treatments are presented in Table 2. The means for the initial height of F4, F5, and F6 were higher than those of F1 and F3. F6 showed a statistically significant difference (*p* < 0.05) in weight when compared to the others. After being subjected to the baking process, F6 (industrialized/plant-based) continued to be the treatment with the highest final height, showing a statistically significant difference (*p* ≤ 0.05) compared to the other treatments.

In a Brazilian study by Higuera et al. [7], the authors mentioned the difference in weight values; according to them, the initial and final weight were less pronounced for the animal protein samples compared to the weight of the plant-based burgers, which can be justified by the number of ingredients used in the formulation of plant-based products.

With regard to the comparison between the initial and final weight of burgers already available on the market and those in this study, De Marchi et al. [32] presented data regarding the initial weight of defrosted burgers, with a mean of 115.09 g (n = 27 products) for meat analogs products and a mean of 152.58 g (n = 24 products) for burgers of animal origin.

F4 (50% GBB) had the highest weight and diameter values before and after baking, indicating better mass retention and dimensional stability. This trend may be related to the balanced presence of teff and chickpeas, which contributed to greater matrix cohesion and moisture retention. F6, with lower weight and greater structural reduction, possibly reflects the use of more processed ingredients with lower retention capacity. The more pronounced reduction in height in the treatments with higher GBB content (F1 and F2) can be explained by the lower heat resistance of the isolated biomass.

The average values for the initial diameters showed that F4 showed a statistically significant difference (*p* ≤ 0.05) when compared to the other treatments; only F2 and F3 did not show a statistically significant difference (*p* > 0.05) between groups. Regarding the final diameter results, F4 showed the highest mean compared to F6 (industrialized/plant-based), with the lowest mean value, both showing a statistically significant difference (*p* ≤ 0.05).

Similar data for diameters were presented in the study by Samard et al. [33], in which the authors reported changes in the diameters of plant-based burgers; according to the authors, this difference was statistically significant (*p* < 0.05) and lower than those of the meat-based burger.

Samard et al. [33] presented data showing that meatless burgers demonstrated a statistically significant difference (*p* ≤ 0.05) when compared to meat burgers. For the authors, the reduction in the shapes of the plant-based burgers after cooking was close to that of the raw burgers before cooking when compared to the means of the animal-origin meat burgers.

**Figure 2:**
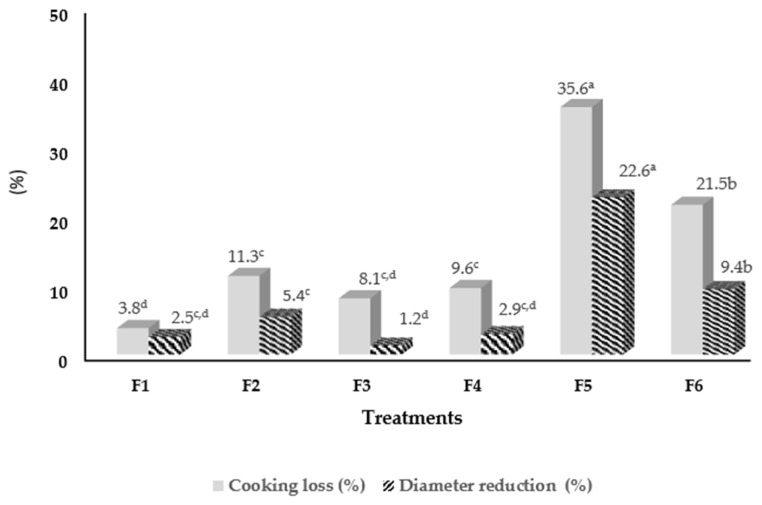
Percentage of cooking loss (%) and diameter reduction (%) in the evaluated burgers. ^abcd^ Different superscript letters in each column indicate a significant difference (*p* ≤ 0.05) for the treatments. Source: study data.

The physical results presented in Figure 2 complement Table 2, showing the percentage of cooking loss and the reduction in diameter of all the burgers after baking. It can be noticed that F5 (industrialized/meat) showed the highest losses, reflecting a lower capacity to retain moisture and fat during heat processing. On the other hand, formulations F1 and F4, with a higher proportion of green banana biomass (GBB), teff, and chickpeas, showed better dimensional stability and lower losses of water and fat. These findings are in accordance with studies by Samard et al. [33] and Pietrasik et al. [34], who highlighted the structural and compositional differences between plant-based formulations during cooking.

According to Samard et al. [33], post-harvest structural modification is influenced by water retention, which alters the values of diameter and height, important properties assessed to determine reduced juiciness during the cooking process. Furthermore, Pietrasik et al. [34] described that burgers with non-meat ingredients, such as cereal flour, plant-based fibers, or starches, are widely used as fillers, binders, and extenders to improve the texture and water and fat retention capacity of plant proteins, creating meat-like matrices.

In a Canadian study by Pietrasik et al. [34], the results of their physical analyses also showed a difference between plant-based products and animal meat products. The authors also highlighted the perception of a reduction in the post-supply process. According to them, the amount of liquid released during the thermal processing of meat products is mainly linked to the retention of fat and water and is generally related to the ability of the protein matrix to retain these elements.

Table 3 presents the means and standard deviation in the analysis of water retention capacity; F3 and F1 showed the highest means for WRC, the industrialized/meat and plant-based products showed the lowest means for WRC, and none of the treatments showed statistically significant differences (*p* < 0.05) between groups.

The color parameters (L*, a*, and b*) and firmness are presented in Table 3 with means and standard deviations for the different treatments. F1 and F6 showed statistically significant differences (*p* ≤ 0.05) between groups. F1 was the treatment that showed the highest mean for (L*), indicating that this treatment has a lighter color than the other treatments, especially compared to F6 (industrialized/plant-based), which had a lower mean for (L*). This can be explained by the use of beetroot powder and charcoal dye described on the product label.

In a Brazilian study by Santos et al. [35], the authors produced two types of hybrid burgers, one enriched with GBB and the other with passion fruit peel biomass. The results showed a statistically significant difference (*p* ≤ 0.05), evidenced by higher L* values (47.81 and 54.63, respectively). According to the same authors, this situation can be attributed both to the presence of GBB, whose color tends to be lighter, and to the use of biomass from the passion fruit epicarp, characterized by its intrinsically whiter coloration.

In Spain, a study carried out by Botella-Martinez et al. [36], the authors developed four treatments of plant-based burgers using gelled emulsions as a source of fat and beet juice as a colorant. The results showed that the PBCCh formulation (chia-gelled and fresh beet juice) had a higher mean of L* (34.87) when compared to PBFH (candy-gelled and fresh beet juice) (33.03), which had a lower mean of L* in the study.

An Italian study by De Marchi et al. [32] compared meat-based and plant-based burgers. In the results for L*, the means of meat and plant-based burgers were 44.89 and 47.99, respectively, which shows that their results are similar to those presented in this study.

Regarding the color for (a*), F6 (industrialized/plant-based) was the treatment that presented the highest mean (14.00) when compared to F1, which presented the lowest mean (2.33). The two treatments presented statistically significant differences (*p* ≤ 0.05) between each other and among the other treatments.

The study by Botella-Martinez et al. [36] obtained the highest mean between PBFCh (chia-gelled and fresh beet juice) and PBFH (hemp-gelled and fresh beet juice) for (a*) (13.18 and 12.21); minimum means were presented by PBCCh (chia-gelled and commercial beet juice) and PBCH (hemp-gelled and commercial beet juice) (7.92 and 7.88), respectively.

The results reported by De Marchi et al. [32] on component (a*) presented a mean for the meat burger of 19.82 and 16.83 for the meat analog.

Table 3 also shows the means for color (b*), showing that F1 had the highest mean (22.55), unlike F5, which had the lowest mean (12.98), demonstrating a statistically significant difference (*p* < 0.05) between them. Botella-Martinez et al. [36] presented the maximum mean for color (b*) in the PBCH treatment (hemp-gelled and commercial beet juice) with a value of 9.72, and the minimum was presented by PBFH (hemp-gelled and fresh beet juice), with a value of 8.14.

In the results presented in the study by De Marchi et al. [32] on component (b*), the authors reported means for beef burgers (14.46) and for plant-based burgers (11.21). The importance of the parameter color and its acceptable range for the difference between plant-based burgers and the standard (meat burger) can be noticed by comparing the color values between the GBB treatments and the industrialized/meat. F1 (100% GBB) presented values of 14.70—that is, a completely different tone from that presented by F5, which can be justified by its base. F3 and F4 obtained extremely notable differences (7.57 and 7.60, respectively). The industrialized plant-based treatment also differed from F5, the meat standard (11.16).

Firmness is an important parameter to evaluate. The values indicate the maximum force required to penetrate the burger samples. The firmness of the GBB burgers, along with the industrialized ones (meat and plant-based), is presented in Table 2. The results showed that F5 (industrialized/meat) presented a value of 9.112.68, demonstrating a statistically significant difference (*p* ≤ 0.05) when compared to all other treatments, which presented lower means. F1, F2, F3, F4, and F6 did not show statistically significant differences (*p* > 0.05) between groups.

In a study conducted in the United States by Kim et al. [37], the authors evaluated the texture of meat analogs formulated with legume proteins and a high moisture content in addition to soy concentrate and soy isolate. In their results, the authors pointed out that legume proteins containing a high moisture content significantly affect firmness attributes, affecting cohesiveness of C1 and T3 (4.0–7.2, respectively), firmness of C1 and T3 (8.3–3.9, respectively), and with the elasticity of treatments C1 and T3 (8.0–4.3, respectively). All the results described showed a statistically significant difference (*p* < 0.0001) between groups.

Table 4 presents the results of elasticity, cohesiveness, resilience, chewiness, and gumminess (texture analyses). The elasticity values range between 1.08 and 1.38; F1, F2, F3, and F4 present similar values and are grouped in the same statistical category, indicating that there is no significant difference (*p* > 0.05) between groups. On the other hand, F5 and F6 showed a statistically significant difference (*p* ≤ 0.05), suggesting that these formulations are less elastic, which may impact the texture and palatability of the product. Regarding cohesiveness, only the F5 (industrialized/meat) showed a statistically significant difference (*p* ≤ 0.05) compared to the other treatments.

The resilience results for F5 (meat), with significantly higher prominence, indicate that it is the most resilient formulation, capable of recovering its shape after being deformed. In contrast, F6 (industrialized/plant-based) has the lowest mean, which can negatively impact durability and structure during consumption. On the other hand, GBB treatments did not show a statistically significant difference between groups (*p* > 0.05).

The chewiness values showed that F5 presented a statistically significant difference (*p* ≤ 0.05) when compared to the others. F2 and F3 were not statistically different from each other, just as F1 and F6 (industrialized/plant-based) did not show any statistical difference between them.

The results on gumminess of F1, F2, F3, F4, and F6 had similar values (*p* > 0.05). F5 (industrialized/plant-based) showed a statistically significant difference (*p* ≤ 0.05) when compared to the other treatments, and F4 stood out with the highest mean, showing greater gumminess, which may suggest a more desirable texture. F5 (industrialized/meat), although it has superior chewiness, presented lower gumminess, which may influence the perception of the product’s texture.

### Chemical Analyses

Table 5 shows the results on the means and standard deviation of the proximate composition, Kcal, and pH analyses of F1, F2, F3, and F4, meat analogs made with GBB associated with teff and chickpea, in addition to industrialized treatments F5 and F6 (meat- and plant-based).

The results for moisture showed that F1, F3, and F4 were the treatments with the highest mean values, without presenting statistically significant differences (*p* > 0.05)between groups, indicating similarity in the moisture contents of these treatments. On the other hand, F2, F5, and F6 showed statistically significant differences (*p* ≤ 0.05) between groups. F5 and F6 presented the lowest moisture content, suggesting that the industrialization process or the specific composition of these burgers leads to a considerable loss of moisture compared to GBB burgers; this moisture reduction can influence their juiciness.

In a Brazilian study carried out by Lima et al. [38], the authors prepared two burgers with textured cashew almond concentrate and soy protein. The treatments with cashew almond concentrate had a higher moisture content (71.4%) compared to the burger with soy protein (70.3%); these results were higher than all the data found in the four treatments with GBB and industrialized/plant-based, in which the mean value was 61.42% (F4).

In this study, all the results for moisture were lower than those reported by the UK study carried out by Latunde-Dada et al. [39], in which the moisture content of cooked burgers ranged from 54.1% (meat) to 75.2% (plant-based). These findings draw attention to the care that must be established in the production and storage of meat analog burgers, as high moisture values in foods can promote the growth of microorganisms, including pathogens such as *Salmonella*, *E. coli*, and *Listeria*, increasing the risk of foodborne diseases.

Based on the results presented in Table 5, ash values did not show a statistically significant difference (*p* > 0.05) between groups. Different results were found in the study by De Marchi et al. [32], in which the authors compared the composition of meat-based and plant-based burgers. The ash content results were 2.52% (plant-based) and 1.79% (animal-based). According to the authors, their results showed statistically significant differences (*p* < 0.05).

In the study by Lima et al. [38], the results for burgers with cashew almond concentrate and soy protein showed mean ash values of 1.3% and 2.1%, respectively. The ash values in the study by Chilón-Llico et al. [40] were similar to those in this research. The treatments had the following means: quinoa (2.50%), lupine (2.30%), and corn (2.20%). According to the same authors, these values did not show a statistically significant difference (*p* >0.05) between groups.

The results of the protein analysis showed different levels of this nutrient in the evaluated burgers. F5 (industrialized/meat) showed a higher mean and a statistically significant difference (*p* ≤ 0.05) when compared to F1, F2, F3, and F4—GBB treatments. F6 (industrialized/plant-based) did not show a statistically significant difference (*p* > 0.05) compared to F5 (industrialized/meat), which can be justified by the addition of textured soy protein, isolated soy protein, and pea protein according to the product labeling. This addition certainly contributed to increasing the protein content of the product.

The minimum values found in this study were higher than those reported by Lima et al. [38]; burgers with cashew almond concentrate and the other with soy protein had mean values for protein of 7.1 and 6.7%. Interesting findings were presented by Chilón-Llico et al. [40], in which the Peruvian authors evaluated plant-based burgers and obtained the following results: 17.63% for white quinoa, 61.44% for lupine, and 8.15% for corn. The result presented for protein in the treatment with corn is in accordance with this study; the others were superior in protein values.

Table 5 presents the means and standard deviations of lipid contents, where F6 exhibited the lowest value and F5 exhibited the highest, with these values being statistically different from each other (*p* ≤ 0.05). The four treatments with GBB showed no statistically significant differences between groups (*p* > 0.05). The reduced lipid content observed in the plant-based treatments suggests a product suitable for daily consumption, considering that a high lipid content in the diet can be associated with health complications.

In the study by Lima et al. [38], the plant-based burgers with cashew almond concentrate and the other with soy protein had lower values for plant-based burgers than in this study, with means for lipids of 1.5 and 1.3%, respectively. In the study by Chilón-Llico et al. [40], the lipid values of plant-based burgers were higher than in this study with GBB, with the mean of white quinoa being 6.76%, that of lupine being 18.29%, and that of corn being 4.58%. These values showed a statistically significant difference (*p* < 0.05) between the treatments.

The results obtained for the carbohydrates presented in Table 5 indicated that treatments F1, F2, F3, and F4 had a higher carbohydrate content compared to F5, with a statistically significant difference (*p* ≤ 0.05). These results are justified by the basic ingredients of these treatments; for example, GBB has a high resistant starch content. Moreover, the higher presence of carbohydrates in plant-based burgers can be promising, providing an interesting source of dietary fiber found in grains and cereals used in these products.

The study by Lima et al. [38] investigated two types of plant-based burgers, one with cashew almond concentrate and the other with soy protein. The results reported by the study for carbohydrates were lower than in this research, with means of 19.6 and 18.7%, respectively. According to the study conducted by Chilón-Llico et al. [40], the carbohydrates obtained in the treatment with white quinoa had a mean of 73.03%, that of lupine was 17.95%, and that of corn was 85.08%. According to the authors, these results showed a statistically significant difference (*p* ≤ 0.05) between groups.

The results presented for kilocalories (Kcal) complement the previous analyses. F5 and F4 did not show a statistical difference between them, indicating a similarity in their caloric values; however, F1, F2, F3, and F6 showed statistically significant differences (*p* ≤ 0.05) when compared to F5. The analysis by Lima et al. [38] showed two types of plant-based burgers, one with cashew almond concentrate and the other with soy protein; their findings for kilocalories were lower (115.4 and 118 kcal), respectively. As reported by the authors of this study, the results showed a statistically significant difference (*p* ≤ 0.05) between burgers.

pH values are described in Table 5. F6 (industrialized/plant-based) showed the highest mean, demonstrating a statistically significant difference (*p* ≤ 0.05) between the treatments. On the other hand, F5 (industrialized/meat) did not show a statistically significant difference between F1, F3, and F4. In a Korean study by Bakhsh et al. [41], lower values were described for beef hamburgers (5.62%) and (6.19%) beef analog burgers. According to the authors, their results showed a statistically significant difference (*p* ≤ 0.05) between groups.

Dietary fiber has been recognized as an important part of a complete diet; its use and its addition into meat products are gaining importance day by day [42]. The incorporation of fibers in meat products has become increasingly common, thanks to their various functional properties, such as water retention, reduction in loss during cooking, lubrication, texture modification, and neutral flavor [42]. Dietary fiber comprises plant elements that are neither digested nor absorbed in the small intestine. These components include non-starchy polysaccharides and oligosaccharides, such as cellulose present in plants and seed extracts [32]. From this perspective, fiber analysis was not performed for F5 (meat-based burger) to focus on the evaluation of fiber content among the plant-based formulations. The results are presented in Table 6. 

Table 6 presents the means and standard deviations for total fiber content (%) of the baked burgers formulated with different proportions of green banana biomass (F1, F2, F3, F4, and F6—industrialized/plant-based).

The results of total fibers demonstrated a statistically significant difference (*p* ≤ 0.05) among F6 (industrialized/plant-based) and the other treatments. The treatments with GBB showed no statistically significant differences (*p* > 0.05) among themselves. The total fiber content in F6 (industrialized/plant-based) is higher than that in GBB treatments, which can be explained by the presence of ingredients with high dietary fiber content, such as textured soy protein and pea protein. According to Kyriakopoulou et al. [43], these ingredients (textured soy protein and pea protein) are processed in a way that preserves or even concentrates the fiber fraction of the original raw material.

The insoluble fiber levels (%) presented in Table 6 showed a constant behavior among the treatments (F1, F2, F3, F4, and F6), with no statistical differences (*p* > 0.05) between groups. The soluble fiber results showed statistically significant differences (*p* ≤ 0.05) between F1 and F6. The study by Curtain and Grafenauer [44] provided an overview of plant-based meat substitutes currently found on Australian supermarket shelves, detailing their nutritional composition compared to animal-based products (burgers, sausages, and ground meat).

The total dietary fiber content of plant-based burgers was reported to be 5.3%, which aligns with the results described in Table 6 for F6 (industrialized/plant-based). De Marchi et al. [32] from Italy compared the nutritional composition of meat-based burgers (MBB) and plant-based burgers (PBB) available in supermarkets across the European Union. According to the authors, MBB contained 0.74% total fiber, while the PBB had 4.27%, similar to F4 GBB, which showed a mean of 4.14% for total fiber.

In a study by He et al. [45], the authors evaluated several industrialized plant-based burgers sold in Canada. Among the evaluated products, the results for total fibers of the six treatments were (1.77%, 2.65%, 3.41%, 3.54%, 4.00%, and 5.31%). These results corroborate the findings of this study, demonstrating that BBV burgers comply with dietary fiber standards.

Internationally, foods can be classified as high in fiber when they contain at least 5 g of fiber per portion according to the World Health Organization and European guidelines [46]. In Brazil, the Anvisa- a National Health Surveillance Agency [47] established the minimum that a food or preparation must contain. Anvisa is in accordance with the technical regulation of the Ministry of Health (Ordinance n°. 27/98), which determines that a ready-to-consume solid product is considered a source of fiber if it contains a minimum of 3g of fiber per 100g of the product [48].

In this sense, it can be stated that all the burgers made with BBV met these recommendations, being among the products characterized as a source of dietary fiber.

In general, the results demonstrate the promising technological performance of GBB, particularly when used as a partial replacement with teff and chickpea, as observed in F4. Its moisture retention, structure stabilization, and fiber enrichment highlight its innovative potential in plant-based product development. Unlike plant proteins more widely used, such as soy and pea isolates, GBB offers a more natural matrix with additional nutritional benefits, which supports its application in clean-label meat analog formulations.

## 4. Conclusions

The development of meat analog burgers, using GBB in combination with teff and chickpea, was an innovative proposal, and some challenges were surpassed, especially texture, since GBB is easy to handle and has a soft texture.

The physical parameters for weight, diameter, and height (initial and final) of GBB burgers showed that F4 was the most robust treatment, with few changes between the initial and final parameters to which it was submitted. F5 (industrialized/meat) showed the highest losses for diameter reduction and cooking loss. The firmness analyses showed that F3 and F4 came closest to F5’s (industrialized/meat) characteristics, surpassing F6 (industrialized plant-based) treatment.

The textural properties of the burgers revealed variations among the formulations, especially in terms of cohesiveness, resilience, chewiness, and gumminess. F4 stood out for presenting the highest means in these parameters, showing that this product combines desirable characteristics of firmness with juiciness, resulting in a more balanced texture that is pleasant to consume.

Regarding the chemical parameters, F3 and F4 were the most favorable treatments because, in the analysis of proximate composition, they showed promising results for proteins, lipids, and carbohydrates; regarding ash values, they showed similarities between treatments. For energy, F1, F2, and F3 were the treatments with the lowest means, while F4 and F5 presented the highest values. Regarding the results for pH, F1, F3, and F4 burgers were similar to F5 (industrialized/meat).

The burgers made with GBB showed fiber values above 3g/100g of product, which proved promising, especially regarding total and insoluble fibers. These results indicate that GBB has good potential to be used in plant-based burgers, offering interesting levels of dietary fiber.

Thus, it can be concluded that the combination of GBB with teff and chickpea was feasible for making meat analog burgers, highlighting F4, which consisted of 50% GBB, 25% teff, and 25% chickpea. This treatment was the one that best met the chemical and technological quality parameters, proving to be the most balanced and efficient formulation.

Future studies performed by our research group should evaluate sensory acceptability, shelf-life stability, and consumer perception to support the product’s applicability in the market.

## Figures and Tables

**Table 1:** Ingredients used for the formulation of plant-based burgers.

Ingredients	Formulations (%)			
	F1 (100%)	F2 (75%)	F3 (60%)	F4 (50%)
Green banana biomass (GBB)	100	75	60	50
Brown teff flour	-	12.5	20	25
Canned chickpeas	-	12.5	20	25
Chickpeas flour	6	6	6	6
Chickpeas aquafaba	5	5	5	5
Dried textured soy protein	20	20	20	20
Ground black pepper	0.02	0.02	0.02	0.02
Garlic in natura	1.0	1.0	1.0	1.0
Onion in natura	1.0	1.0	1.0	1.0
Chives and parsley in natura	2.0	2.0	2.0	2.0
Refined sea salt	1.8	1.8	1.8	1.8
Soybean oil	6	6	6	6
Smoked paprika	0.20	0.20	0.20	0.20
Dried seasoning (garlic, onion, rosemary, cilantro, parsley)	1.8	1.8	1.8	1.8
Crushed ice	10	10	10	10

Source: Adapted from De Oliveira Rosa; Lobato [21]. According to the products’ labels, F5 (meat-based) contains beef, water, salt, and ascorbic acid (INS 300), while F6 (plant-based) is composed of water, soy and pea protein derivatives, vegetable fat, beetroot powder, natural flavoring, methylcellulose, and coloring agents. Both products were purchased from a local supermarket in the respective meat and plant-based product sections.

**Table 2:** Means and standard deviation of the physical analyses of burgers.

Physical Analyses						
	Initial Weight (g)	FinalWeight (g)	Initial Diameter (cm)	Final Diameter (cm)	Initial Height (cm)	Final Height (cm)
F1 F2 F3 F4 F5 F6	184.33 ± 2.51 ^c^ 206.66 ±5.85 ^b^ 214.66 ± 1.52 ^ab^ 223.00 ± 11.35 ^a^ 169.25 ± 3.23 ^c^ 116.22 ± 0.94 ^d^	177.33 ± 2.08 ^c^ 183.33 ± 8.08 ^bc^ 197.33 ± 2.51 ^ab^ 201.66 ± 11.01 ^a^ 108.91 ± 7.15 ^d^ 91.18 ±2.48 ^d^	10.91 ± 0.27 ^c^ 11.78 ± 0.37 ^b^ 11.42 ± 0.26 ^bc^ 12.33 ± 0.56 ^a^ 11.06 ± 0.33 ^c^ 8.57 ± 0.27 ^d^	10.65 ± 0.16 ^c^ 11.17 ± 0.30 ^b^ 11.27 ± 0.23 ^b^ 11.96 ± 0.58 ^a^ 8.55 ±0.22 ^d^ 7.76 ±0.15 ^e^	1.72 ±0.08 ^d^ 1.96 ± 0.12 ^bc^ 1.80 ± 0.14 ^cd^ 2.04 ± 0.11 ^b^ 2.11 ± 0.15 ^b^ 2.42 ± 0.09 ^a^	1.48 ± 0.13 ^c^ 1.46 ± 0.07 ^c^ 1.44 ± 0.07 ^c^ 1.57 ± 0.12 ^bc^ 1.68 ± 0.09 ^b^ 2.10 ± 0.15 ^a^

^abcde^ Different superscript letters in each column indicate a significant difference (*p* ≤ 0.05) for the treatments. F1—100%; F2—75%; F3—60%; F4—50% green banana biomass, F5—industrialized/meat; F6—industrialized/plant-based.

**Table 3:** Mean and standard deviation of water retention capacity, color, and firmness of the meat analogs and industrialized (meat and plant-based burgers) treatments.

	Water Retention Capacity—WRC (%)		Color		Firmness (g)
		L*	a*	b*	
F1 F2 F3 F4 F5 F6	35.94 ± 13.10 ^a^ 30.18 ± 0.26 ^a^ 39.95 ± 13.87 ^a^ 32.57 ± 0.97 ^a^ 28.85 ± 0.53 ^a^ 27.19 ± 2.37 ^a^	53.11 ± 1.85 ^a^ 45.77 ± 1.28 ^b^ 44.87 ± 1.23 ^b^ 44.50 ± 0.87 ^b^ 44.10 ± 4.60 ^b^ 34.40 ± 1.14 ^c^	2.33 ± 0.50 ^d^ 6.09 ± 0.58 ^c^ 5.99 ± 0.54 ^c^ 6.07 ± 0.29 ^c^ 8.92 ± 1.11 ^b^ 14.00 ± 1.54 ^a^	22.55 ± 1.68 ^a^ 22.30 ± 1.39 ^a^ 19.92 ± 0.97 ^b^ 20.01 ± 0.55 ^b^ 12.98 ± 1.62 ^d^ 15.16 ± 0.92 ^c^	1133.52 ± 192.22 ^b^ 1978.42 ± 501.38 ^b^ 2096.81 ± 718.17 ^b^ 2512.98 ± 329.19 ^b^ 9112.68 ± 1936.30 ^a^ 1485.49 ± 452.95 ^b^

^abcd^ Different superscript letters in each column indicate a significant difference (*p* ≤ 0.05) for the treatments. F1—100%; F2—75%; F3—60%; F4—50% green banana biomass, F5—industrialized/meat; F6—industrialized/plant-based.

**Table 4:** Means and standard deviation of the burger’s treatments regarding elasticity, cohesiveness, resilience, chewiness, and gumminess.

	Elasticity (g)	Cohesiveness (g)	Resilience (g)	Chewiness (kgf)	Gumminess (kgf)
F1 F2 F3 F4 F5 F6	1.38 ± 0.011 ^a^ 1.36 ± 0.008 ^a^ 1.37 ± 0.178 ^a^ 1.36 ± 0.004 ^a^ 1.08 ± 0.011 ^c^ 1.19 ± 0.020 ^b^	1.14 ± 0.127 ^a^ 1.04 ± 0.075 ^a^ 1.11 ± 0.060 ^a^ 1.16 ± 0.080 ^a^ 0.77 ± 0.054 ^b^ 1.08 ± 0.125 ^a^	3644.80 ± 577.25 ^b^ 6296.80 ± 1762.60 ^b^ 5908.40 ± 1449.98 ^b^ 6299.00 ± 1056.75 ^b^ 21263.40 ± 5046.16 ^a^ 2811.40 ± 961.81 ^b^	1797.20 ± 212.56 ^d^ 2815.00 ± 219.09 ^c^ 3206.00 ± 182.22 ^c^ 3994.00 ± 290.63 ^b^ 7649.80 ± 619.48 ^a^ 1931.80 ± 236.93 ^d^	129,655.13 ± 14552.96 ^a^ 118,174.31 ± 8589.67 ^a^ 126,451.95 ± 6790.30 ^a^ 132,141.63 ± 9215.50 ^a^ 87704.28 ± 6158.32 ^b^ 123,267.28 ± 14526.62 ^a^

^abcd^ Different superscript letters in each column indicate a significant difference (*p* ≤ 0.05) for the treatments. F1—100%; F2—75%; F3—60%; F4—50% green banana biomass, F5—industrialized/meat; F6—industrialized/plant-based.

**Table 5:** Means and standard deviation of the proximate composition, Kcal, and pH analyses of the burger treatments.

Chemical Parameters							
	Moisture (%)	Ash (%)	Proteins (%)	Lipids (%)	CHO (%)	Kcal	pH (%)
F1 F2 F3 F4 F5 F6	61.91 ± 0.55 ^a^ 58.53 ± 0.29 ^b^ 60.09 ± 0.73 ^ab^ 61.42 ± 0.63 ^a^ 45.09 ± 0.28 ^d^ 55.40 ± 1.70 ^c^	2.10 ± 0.13 ^a^ 2.45 ± 0.06 ^a^ 2.18 ± 0.08 ^a^ 2.25 ± 0.02 ^a^ 3.62 ± 4.71 ^a^ 3.07 ± 0.16 ^a^	8.75 ± 1.20 ^c^ 8.13 ± 1.02 ^c^ 10.29 ± 4.97 ^bc^ 10.25 ± 0.21 ^bc^ 31.53 ± 10.42 ^a^ 23.10 ± 4.05 ^ab^	3.58 ± 0.24 ^b^ 2.32 ± 0.63 ^b^ 3.07 ± 0.55 ^b^ 3.19 ± 2.10 ^b^ 16.04 ± 5.64 ^a^ 2.60 ± 1.24 ^b^	23.63 ± 1.27 ^a^ 28.55 ± 1.36 ^a^ 24.36 ± 4.63 ^a^ 22.87 ± 2.06 ^a^ 3.70 ± 11.93 ^b^ 12.59 ± 6.33 ^ab^	161.80 ± 2.89 ^b^ 167.73 ± 4.06 ^b^ 166.23 ± 5.88 ^b^ 254.63 ± 82.36 ^ab^ 285.30 ± 15.72 ^a^ 174.30 ± 1.73 ^b^	7.62 ± 0.03 ^b^ 7.51 ± 0.02 ^c^ 7.58 ± 0.03 ^bc^ 7.61 ± 0.02 ^b^ 7.57 ± 0.03 ^b^ 7.93 ± 0.02 ^a^

^abcd^ Different superscript letters in each column indicate a significant difference (*p* ≤ 0.05) for the treatments. F1—100%; F2—75%; F3—60%; F4—50% green banana biomass, F5—industrialized/meat; F6—industrialized/plant-based.

**Table 6:** Results of the means, standard deviation, and coefficient of variation of the fiber analysis results of green banana biomass hamburgers.

Fibers			Treatments			
	F1 (%)	F2 (%)	F3 (%)	F4 (%)	F5 (%)	F6 (%)
Total fibers Insoluble fibers Soluble fibers	3.62 ± 0.08 ^b^ 3.03 ± 0.04 ^a^ 0.59 ± 0.03 ^b^	3.74 ± 0.08 ^b^ 3.03 ± 0.16 ^a^ 0.71 ± 0.08 ^ab^	3.97 ± 0.01 ^b^ 3.19 ± 0.05 ^a^ 0.78 ± 0.07 ^ab^	4.15 ± 0.61 ^b^ 3.55 ± 0.51 ^a^ 0.59 ± 0.10 ^b^	-- -- --	5.69 ± 0.14 ^a^ 3.25 ± 0.35 ^a^ 1.14 ± 0.21 ^a^

^ab^ Different superscript letters in each column indicate a significant difference (*p* ≤ 0.05) for the treatments. F1—100%; F2—75%; F3—60%; F4—50% green banana biomass, F5—industrialized/meat; F6—industrialized/plant-based.

## Data Availability

The original contributions presented in the study are included in the article, further inquiries can be directed to the corresponding author.
